# Ethical Challenges and Governance Strategies for Microphysiological Systems Technology

**DOI:** 10.3390/biology15131092

**Published:** 2026-07-07

**Authors:** Manman Zhao, Tian Lin, Ruiqiu Zhang, Haodong Zhong, Qianyi Niu, Xiaobing Zhou, Qingli Wang

**Affiliations:** 1 National Center for Safety Evaluation of Drugs, National Institutes for Food and Drug Control, Beijing 102600, China; 2 State Key Laboratory of Natural Medicines, China Pharmaceutical University, Nanjing 211198, China; 3 National Institutes for Food and Drug Control, Chinese Academy of Medical Sciences & Peking Union Medical College, Beijing 100730, China; 4 School of Pharmacy, Shenyang Pharmaceutical University, Shenyang 110016, China

**Keywords:** microphysiological systems, organ-on-a-chip, organoid, ethical challenges, data privacy, governance strategies

## Abstract

Microphysiological Systems (MPS) have emerged as a transformative platform in biomedical research, enabling the investigation of disease mechanisms, drug screening, and toxicity prediction by closely simulating human physiological functions. However, the rapid advancement of MPS technology has raised a series of complex ethical challenges. These include the sourcing and application of human-derived stem cells, the protection of donors’ personal and genetic data, the potential for brain organoids to develop consciousness-like characteristics, and the challenges to species boundaries posed by human–animal chimera research. Meanwhile, although regulatory authorities encourage innovation, specialized certification standards and ethical review guidelines for MPS are yet to be fully established. The lack of technical standardization and a coherent ethical governance framework remain a major bottleneck hindering the broader application and industrialization of MPS. This review systematically outlines the key ethical issues facing MPS, compares the evolution and differences in international ethical regulatory frameworks, and discusses strategies for addressing these challenges—including the establishment of dynamic ethical governance mechanisms, harmonization of international standards, and the promotion of benefit-sharing and public engagement. Finally, we highlight the need to develop a scientific, unified, and actionable ethical governance system that balances technological innovation with responsible translation, supporting the sustainable development of MPS technology.

## Simple Summary

Microphysiological Systems, new lab tools that mimic human body functions, advance medical research and drug testing yet raise various ethical concerns. These involve the collection and use of human cells, genetic privacy protection, potential risks of lab-grown brain tissues, and controversies over human–animal research. Lacking complete official rules and unified technical standards, the technology is held back from wide adoption and industrialization. This paper addresses major ethical dilemmas, compares global regulations, and puts forward solutions including flexible ethical management, coordinated international standards, public participation and fair benefit sharing. A sound ethical framework balances technological progress and safe use, drives the steady development of Microphysiological Systems, and ultimately benefits public health and society.

## 1. Introduction

MPSs refer to microscale in vitro cell culture platforms that recapitulate key physiological and pathological characteristics by constructing biomimetic microenvironments for cells to mimic functional phenotypes of specific human or animal tissues and organs. In recent years, MPS technology has emerged as a transformative force driving paradigm shifts in biomedical research. Representative models include organ-on-a-chip and organoids, which exhibit revolutionary potential in disease mechanism research, drug screening, personalized medicine, and toxicity assessment [1]. In particular, by integrating precisely controlled microenvironments, multicellular interactions, and programmable perturbation strategies, MPS platforms enable researchers not only to observe biological responses but also to actively dissect causal relationships underlying disease progression and drug responses [2,3,4].

However, with the rapid advancement of MPS technology, challenges related to ethics and legal safeguards have become increasingly prominent. From an ethical perspective, one major concern involves moral boundaries associated with the sourcing of human cells, including issues of informed consent in the acquisition and use of embryonic stem cells (ESCs) and induced pluripotent stem cells (iPSCs). Another relates to emerging ethical risks such as the potential generation of consciousness, altered animal behavior, and the blurring of species boundaries [5]. In terms of data and privacy, genomic, transcriptomic, and other biomarker data generated by MPS may reveal highly individual donor characteristics. Inadequate de-identification or lax regulation could increase the risk of re-identification, thereby exposing personal information. At the policy level, the U.S. Food and Drug Administration (FDA) has begun incorporating organ-on-a-chip and other microphysiological models into its New Approach Methodologies (NAMs) strategy, issuing guidance documents to promote their application in nonclinical drug safety evaluations [6]. On 29 April 2025, China’s Ministry of Science and Technology released the Ethical Guidelines for Human Organoid Research, which established a systematic ethical framework covering cell sourcing, risk-tiered management, ethical review, and data compliance requirements. This policy foundation for MPS research in China imposes heightened compliance demands on researchers [7]. However, significant variations remain across countries and regions in regulatory standards, model validation procedures, ethical review requirements, and compliance pathways. The lack of harmonization in technical validation and ethical oversight frameworks continues to pose substantial barriers to the broad application and industrial translation of MPS technologies.

Therefore, establishing a scientific, unified, and actionable ethical and safety assessment framework is crucial for promoting the healthy translation of microphysiological systems technology between research and clinical practice. This framework must balance ethical value judgments while ensuring equitable benefit distribution and maintaining public trust.

Several scholars have already discussed ethical concerns associated with organoids, organ-on-a-chip systems, and related stem-cell technologies. Existing reviews have primarily focused on specific issues such as stem cell sourcing, donor consent, privacy protection, brain organoid consciousness, and the ethical implications of human–animal chimeras. For example, Mollaki highlighted the ethical challenges associated with microphysiological systems, particularly issues related to informed consent, privacy, and commercialization [8]. Thakar and Fenton discussed the bioethical implications of organ-on-a-chip technologies in modern drug development, emphasizing regulatory acceptance and responsible innovation [5]. In addition, several reports from the National Academies of Sciences, Engineering, and Medicine have examined the ethical governance of neural organoids, transplants, and chimeras.

Despite these valuable contributions, current discussions are often fragmented and tend to focus on individual technologies or isolated ethical issues. A comprehensive analysis integrating organoids, organ-on-a-chip systems, data governance, chimera research, and international regulatory frameworks within the broader context of microphysiological systems remains limited. Moreover, recent regulatory developments, including China’s Ethical Guidelines for Human-Derived Organoid Research (2025) [7] and the growing global adoption of New Approach Methodologies (NAMs), have created new ethical and governance challenges that have not yet been systematically reviewed [9]. Therefore, this review aims to provide an updated and integrated analysis of the ethical challenges and governance strategies associated with MPS technology from both international regulatory and translational perspectives.

To clarify the scope of this review, the major MPS-related models and their applications, advantages, limitations, ethical concerns, and governance considerations are summarized in Table 1.

## 2. Ethical Challenges of Microphysiological Systems

Currently, the ethical controversies surrounding MPS primarily focus on the following aspects: the ethical boundaries of human-derived stem cells in research and application; the privacy of individual genetic information and data security safeguards; whether models such as organoids and organ-on-a-chip possess a certain moral status; and the ethical risks inherent in human–animal chimera research.

### 2.1. Origin and Application Limits of Human Cells

The construction of organoid and organ-on-a-chip models relies on human cell resources with clear origins and controllable quality. Organoids are primarily obtained through differentiation of various human-derived stem cells, such as embryonic stem cells (ESCs), induced pluripotent stem cells (iPSCs), and adult stem cells (ASCs). The biomaterial sources for organ-on-a-chip systems may include primary human cells, organoids, or cell lines, with the former two being the predominant sources.

ESCs are pluripotent cells derived from the inner cell mass of blastocyst-stage embryos. They possess sustained self-renewal capacity and broad differentiation potential under in vitro culture conditions, conferring important application value in complex tissue modeling and regenerative medicine, and making them one of the most essential cellular sources for organoid research. However, obtaining ESCs typically requires isolating the inner cell mass from early-stage human embryos, a process that damages the donor embryo. This has sparked ongoing debates over whether embryos possess ethical and legal personhood status. National stances on this issue vary considerably [10]. For example, Germany’s Embryo Protection Act of 1990 explicitly declares that embryos possess human status from the moment of fertilization. Accordingly, it prohibits embryo donation and any research-related manipulation, including genetic testing or the extraction of totipotent cells capable of developing into various tissues and organs [11]. In contrast, countries such as the United Kingdom, the Netherlands, and Belgium allow certain embryo research within well-defined ethical frameworks. The most representative ethical boundary is the “14-day rule”, which stipulates that embryos may not be cultured in vitro for more than 14 days. However, recent advances in embryo culture technologies and stem cell-based embryo models have prompted renewed debate regarding both the ethical and scientific justification of this limit. For example, the International Society for Stem Cell Research (ISSCR) recommends that research exceeding this threshold should be considered on a case-by-case basis under specialized ethical review and regulatory oversight rather than being categorically excluded [12]. It should be clarified that the 14-day rule mainly regulates the in vitro culture of embryo models. ESC lines are usually derived from morulae or blastocysts at day 4–6 of development, and their applications include organoid construction, disease modeling, drug screening, and regenerative medicine research. Ethical concerns over ESC lines thus rarely relate to extended embryo culture, but center on whether their subsequent differentiation into diverse organoids exceeds donors’ initial informed consent scope [12].

iPSCs are another critical cellular source for MPS. iPSCs are generally generated by reprogramming somatic cells (such as skin fibroblasts or peripheral blood mononuclear cells) via the introduction of specific transcription factors. In the construction of MPS, iPSCs can be applied in multiple modalities: they can be first directionally differentiated into specific somatic cell types and then co-cultured with other cell populations; alternatively, they can be induced to differentiate into organoids, which are further assembled with other cells or organoids to establish sophisticated multi-tissue and multi-organ interaction systems. In terms of differentiation strategies, iPSCs often undergo multi-step differentiation and possess the potential to differentiate into various lineages and distinct cell types. Moreover, cells or organoids derived from iPSC differentiation may be shared and reused across independent research projects. Given the high complexity and versatility in the subsequent applications and scope of use for iPSC-derived materials, the content and scope of informed consent obtained at the initial somatic cell collection stage should be carefully defined to adequately cover foreseeable future research applications. This donor-related feature also distinguishes iPSCs from ESCs from an ethical and regulatory perspective. Although many technical applications of iPSCs, such as multi-lineage differentiation, organoid construction, and disease modeling, can also be achieved using ESCs, iPSCs are usually derived from living donors and retain highly personalized genetic and health-related information. Therefore, iPSC-derived cells and organoids may be associated with identifiable individuals, especially when linked with genomic, clinical, lifestyle, or disease-response data. This raises specific concerns regarding re-identification, personal data protection, secondary use, and re-consent after donation. By contrast, the ethical issues associated with ESCs mainly concern embryo-source ethics, parental consent, and the moral status of embryos, whereas the risk of linking downstream data to a living donor is generally lower.

To address these challenges, the “dynamic consent” mechanism has garnered increasing attention. This approach advocates for donors’ continuous right to information and decision-making throughout the research cycle. Research institutions enhance consent flexibility and donor autonomy by providing platforms such as smartphone applications or dedicated website portals, enabling donors to modify consent terms, update usage purposes, or even withdraw authorization [13]. Such mechanisms improve research transparency and strengthen participants’ sense of control over their own samples. Overall, while the ethical concerns surrounding iPSCs are less severe than those associated with ESCs in terms of “embryo destruction”, numerous issues still require resolution at the institutional, policy, and practical levels regarding donor informed consent, transparency of usage, future commercial and patent prospects, as well as sample and data sharing and privacy security.

However, the application of dynamic consent in MPS research presents additional challenges that extend beyond those encountered in conventional stem cell studies. Unlike many traditional research models, MPS platforms frequently involve long-term expansion of iPSC lines, repeated differentiation into multiple tissue types, integration with biomaterials and microfluidic devices, and reuse across different research projects [13]. Consequently, the scope of the original informed consent may become increasingly difficult to define over time. For example, an iPSC line initially generated for the construction of a cardiac microphysiological model may subsequently be used to develop neural organoids, brain-on-a-chip systems, or multi-organ platforms investigating entirely different disease contexts. Whether such secondary applications remain within the boundaries of the donor’s original authorization remains ethically controversial [13,14]. Furthermore, collaborations among academic institutions, industrial partners, and international research consortia may introduce additional questions regarding data sharing, intellectual property rights, and commercial utilization. Therefore, ethical governance of iPSC-derived MPS should move beyond a one-time consent model and establish scenario-specific, continuously updated consent mechanisms capable of accommodating evolving research objectives and technological applications while preserving donor autonomy and transparency [15].

Primary human cells can retain the natural physiological functions and metabolic characteristics of organs to the greatest extent, serving as the core material for achieving high-precision physiological simulation in organ-on-a-chip systems. Article 1007 of China’s Civil Code explicitly prohibits the sale of human cells, tissues, organs, or remains in any form, which establishes a robust ethical and legal bulwark against the commodification of human biological materials.

Meanwhile, the Regulations on Human Genetic Resources Management and the Measures for Ethical Review of Life Science and Medical Research Involving Humans further emphasize legality, informed consent, and ethical review in the acquisition and use of human-derived materials. The ethical basis of these restrictions is not merely to limit commercial profit, but to prevent the commodification of the human body, protect donor dignity, avoid exploitation of vulnerable populations, and maintain public trust in biomedical research. At the same time, altruistic donation and publicly governed use of human biological materials can also contribute substantially to scientific progress, medical innovation, and societal benefit, even in the absence of direct commercial profit for private entities. Therefore, the central ethical issue is not a simple opposition between commercialization and innovation, but how to ensure that donated human materials are used transparently, fairly, and in ways that respect donor autonomy and promote public benefit.

Under this regulatory and ethical framework, primary human cells—core biological materials for organ-on-a-chip systems—still face practical challenges in supply and standardization. In China, human-derived cells used for research may be obtained from multiple sources, including surgical residual tissues, qualified organ or tissue donations, and minimally invasive samples such as peripheral blood, bone marrow, urine-derived cells, or other somatic cells used for iPSC generation. The limited availability of such sources may be influenced by multiple factors, including strict ethical review requirements, the absence of specialized procurement and processing institutions, variability in sample quality, and the lack of standardized procedures for isolation, purification, characterization, cryopreservation, and quality control. Therefore, the shortage of primary human cells should not be attributed solely to the lack of donor reimbursement or the prohibition of commercial sale. Rather, it reflects a broader governance challenge: how to establish compliant, transparent, and standardized human tissue procurement and cell-processing systems that can support organ-on-a-chip research while preserving the ethical principles of voluntary donation, non-commodification, donor protection, and fair benefit sharing.

Organoid technology, as a significant derivative form of stem cell research, similarly confronts complex ethical and social challenges. Particularly for tumor organoids, patient-derived tumor organoids (PDOs) have emerged as highly valuable three-dimensional models capable of retaining numerous genetic, phenotypic, and functional characteristics of the original tumor. Compared with conventional two-dimensional cell cultures or animal models, PDOs enable studies of tumor biology, drug efficacy, and precision medicine under conditions that more closely recapitulate the physiological state [16]. For example, colorectal cancer organoids retain the genetic and phenotypic features of the matched primary tumors, making them valuable for preclinical drug screening and individualized treatment evaluation, while preserving tumor heterogeneity and specific histopathological traits [17]. Despite these scientific advances, the distinctive features of tumor organoid models also raise nuanced ethical concerns.

First, because PDOs faithfully recapitulate the genetic and molecular signatures of individual patients’ tumors, they generate extensive genomic and multi-omic datasets that may include highly individualizable information [18]. This raises privacy and data governance challenges, as high-resolution sequencing data from organoids—including whole-exome or transcriptome profiles—could potentially be traced back to donors if cross-referenced with other genetic databases, even when de-identified, particularly when coupled with clinical annotations used to interpret therapeutic responses. The risk of re-identification and unintended disclosure of sensitive health information thus heightens the ethical stakes for data protection and consent, especially in broad or longitudinal research programs that reuse organoid lines across multiple projects [19].

Second, different tumor types may pose distinct ethical considerations based on their clinical context and data content. For instance, pancreatic cancer PDOs not only reflect complex intratumoral heterogeneity but often require detailed molecular characterization to inform co-clinical studies, which further amplifies data sensitivity and complicates consent scope [20]. Similarly, breast cancer organoids have been shown to preserve receptor status and mutational landscapes, making them indispensable for hormone-targeted therapy research; yet this same depth of biological fidelity means that donors’ tumor-specific genomic information could become part of shared research datasets [21].

Moreover, tumor organoid platforms are increasingly integrated with emerging technologies—such as co-cultures with immune cells, microfluidic systems, and high-throughput multi-omics—amplifying both their translational potential and ethical complexity [22]. Such integrations can generate rich profiles of tumor–immune interactions and drug response phenotypes that may have clinical relevance or predictive value for specific subpopulations. While this promises to advance precision oncology, it also blurs traditional boundaries between research and clinical care, potentially fostering therapeutic misconceptions if donors expect direct clinical benefit from organoid-based analyses [23].

In China, human biological samples and genetic information used in organoid research are also classified as critical human genetic resources. However, there remains a consensus among academia and industry that current regulations—particularly the Regulations on the Administration of Human Genetic Resources—may not fully apply to or may require special consideration for data derived from organoid studies, given the ambiguity and evolving nature of this emerging field [24]. Although the Ethical Guidelines for Human-Derived Organoid Research emphasize the importance of ethical review and informed consent, and impose special requirements on highly sensitive models like brain organoids, specific operational details—such as the ownership, sharing, and cross-border transfer of data generated from tumor organoid research—remain insufficiently defined. Whether such data should be governed entirely under the same framework as traditional human genetic resources is still under debate, highlighting the need for more detailed and tailored regulatory frameworks [7].

Furthermore, at the commercialization level, the development of tumor organoids has triggered disputes over benefit distribution and intellectual property ownership. As derivative resources originating from patient biological materials, organoids can be used not only for basic research but also to advance drug development and the translation of precision medicine products. However, substantial disagreements persist regarding whether patients should be entitled to certain rights in commercial profits or to what extent they should participate in decision-making. When patient-derived samples directly contribute to the creation of high-value products, research institutions and companies are encouraged to explore equitable benefit-sharing mechanisms to maintain public trust and foster sustained participation in sample donation [25]. At the same time, competition among research institutions and companies over patent applications and intellectual property rights related to organoids and their derivatives may promote short-term technology translation but also risks hindering academic collaboration and limiting open access to research materials and data. Beyond conventional intellectual-property disputes, organoid and stem cell research raises distinctive ethical concerns because patient-derived biological materials may retain long-term scientific and commercial value through continuous expansion, biobanking, and repeated downstream applications. Consequently, ethical questions arise regarding whether donors should be informed about future commercial uses, how benefits generated from donated materials should be shared, and how donor contributions can be acknowledged while preserving broad access to scientific advances. Therefore, the central ethical challenge is not merely balancing intellectual-property protection with public interests, but ensuring fairness, transparency, reciprocity, and public trust throughout the lifecycle of organoid-based innovation.

### 2.2. Privacy Protection of Individual Data

With the widespread application of advanced cellular technologies such as iPSCs in MPS, concerns regarding data security and privacy involving individual genomic information have become increasingly prominent. iPSCs exhibit high individual specificity, and their whole-genome information holds significant value for disease mechanism research and drug screening. However, it may also expose the donor’s genetic characteristics, disease susceptibility, and even familial relationships [26]. Such information is highly sensitive. Even when researchers employ de-identification measures, genomic samples can still be re-identified when cross-referenced with large-scale population databases containing extensive reference genotypes. This risk is particularly pronounced in MPS research, as experiments often require long-term tracking of the functional status and genetic stability of donor-derived cells. These processes may involve frequent genomic sequencing, transcriptomic profiling, and epigenetic mapping, thereby increasing the risk of information leakage [27].

In response to growing concerns over the privacy and ethical governance of genetic and cellular data in MPS research, developed countries in Europe, America, and increasingly China have established distinct regulatory frameworks, each reflecting national priorities and legal traditions. In the United States, governance is sector-specific and multi-layered, with the Health Insurance Portability and Accountability Act (HIPAA) serving as the core legal instrument for medical and research data protection, requiring de-identification of Protected Health Information (PHI) before use in research and exempting such de-identified data from individual authorization requirements [28]. Complementing HIPAA, the NIH Guidelines for Human Stem Cell Research provide explicit guidance on donor informed consent, oversight for hESC and iPSC research, and compliance monitoring through Institutional Review Boards (IRBs) [9]. This framework emphasizes federal funding control, tiered privacy protections, and institutional responsibility for ethical compliance. In contrast, the European Union has implemented a more integrated, data-centric approach. Under the GDPR, genetic and health-related data—including those derived from iPSCs or organoids—are treated as “special categories” requiring explicit informed consent, clearly defined usage purposes, and traceable, auditable data processing throughout the data lifecycle [29]. The general ethical framework established in Opinion No. 32 Ethics of Genome Editing issued by the European Group on Ethics in Science and New Technologies (EGE) provides ethical constraints and guidance for gene editing of iPSCs and their subsequent modelling applications in organoids and organ-on-a-chip systems. This document stipulates that informed consent for genome editing shall be specific, explicit and revocable, with full disclosure of potential risks associated with genetic modification, long-term sample biobanking, secondary research, data sharing and commercialization. It mandates risk–benefit assessments covering off-target effects, genomic instability and tumorigenic potential prior to editing, as well as the establishment of a full-life-cycle traceability and control mechanism for cells and genetic data. Ethical boundaries are defined for sensitive research involving interspecies chimeras and highly complex in vitro human neural models. Meanwhile, material transfer agreements, cross-border data supervision and clauses restricting the commercialization of biological samples are adopted to standardize sample circulation and translational activities.

Meanwhile, China has developed regulatory model for human cellular and organoid research. The Ministry of Science and Technology (MOST), the National Medical Products Administration (NMPA), and the National Science and Technology Ethics Committee collectively oversee compliance with documents such as the Ethical Guidelines for Research on Human Embryonic Stem Cells (2003) [30], the Regulations on the Administration of Human Genetic Resources (2019) [31], and the recently issued Ethical Guidelines for Human Organoid Research (2025) [7]. The recently issued Ethical Guidelines for Research on Human-Derived Organoids (2025) further clarify the governance framework for advanced human-derived biological models in China [7]. The guidelines establish a multi-level oversight system involving research institutions, ethics committees, funding agencies, and relevant governmental authorities. Ethical review is required throughout the research lifecycle, with particular emphasis on project approval, protocol modification, sample reuse, data management, and cross-institutional collaboration. In addition to procedural oversight, the guidelines identify several priority ethical risks, including the sourcing and use of human biological materials, informed consent, protection of personal genetic information, commercialization of organoid-derived resources, and research involving brain organoids or human–animal chimeras. Particular attention is given to preventing the emergence of ethically sensitive applications that may blur species boundaries or raise concerns regarding cognitive functions and moral status. These provisions reflect China’s effort to combine scientific innovation with proactive ethical governance and biosecurity considerations [7]. In Europe, the European Group on Ethics in Science and New Technologies provides ethical guidance for emerging life science technologies, while the UK Human Fertilisation and Embryology Act and the Human Fertilisation and Embryology Authority provide a regulatory basis for embryo research and the 14-day rule [32,33].

China’s framework combines institutional self-review with mandatory governmental registration, highlighting continuous oversight, social responsibility, and explicit requirements for informed consent and cross-border data governance. In practice, cross-border governance of human genetic and organoid-related data in China is primarily governed by the Regulations on the Administration of Human Genetic Resources (2019) [31], together with the Data Security Law and the Personal Information Protection Law. These regulations require that the provision of certain human genetic resource information to foreign entities be subject to regulatory review or filing procedures and impose additional safeguards for data involving national security, public interests, or sensitive personal information [34,35]. From an ethical perspective, such measures seek to strengthen donor autonomy, privacy protection, and national biosecurity. At the same time, they may generate tensions with other important values, including freedom of scientific research, international collaboration, open science, and the equitable sharing of benefits arising from global biomedical innovation. Therefore, an important challenge for future governance is how to balance data sovereignty and individual protection with the need for responsible cross-border scientific cooperation that advances global health and societal benefit.

Across these jurisdictions, shared ethical goals—protecting donor autonomy, ensuring data privacy, and promoting responsible scientific innovation—emerge despite differing legal instruments, governance structures, and enforcement mechanisms. Notably, while the U.S. relies on a tiered, research-centric model emphasizing federal funding oversight, Europe prioritizes integrated data protection with precautionary guidance, and China focuses on dual-layer oversight linking institutional responsibility with centralized government review. These similarities and divergences reflect both the technical complexity of MPS research and the evolving nature of ethical and legal norms across global contexts.

Although the United States, Europe, and China share common ethical objectives—including the protection of donor autonomy, privacy, and responsible scientific innovation—their regulatory approaches are rooted in distinct governance philosophies. The U.S. framework is primarily innovation-oriented and risk-based, emphasizing institutional responsibility, federal funding oversight, and flexible governance mechanisms that facilitate technological development while managing ethical risks [28,29]. In contrast, the European regulatory system is grounded in the protection of fundamental individual rights, particularly privacy, autonomy, and data sovereignty. Consequently, regulations such as the GDPR impose stringent requirements on consent, data processing, and cross-border data transfer, reflecting a precautionary approach to emerging biomedical technologies [29,32]. China adopts a governance model that combines ethical oversight with national biosecurity considerations and centralized administrative supervision. Regulations governing human genetic resources and organoid research emphasize social responsibility, state oversight, and the protection of strategic biological resources [30,31]. While this approach strengthens accountability and regulatory control, it may also create challenges related to administrative complexity, approval timelines, and international collaboration. Similarly, the European model faces difficulties in balancing privacy protection with data sharing and scientific openness, whereas the U.S. system has been criticized for potential inconsistencies arising from decentralized institutional oversight. These differences illustrate that ethical governance of MPS is shaped not only by technological considerations but also by broader legal traditions, cultural values, and policy priorities. Understanding these underlying governance philosophies is essential for promoting international regulatory harmonization and mutual recognition in the future. The major international ethical regulatory frameworks discussed above are summarized in Table 2.

### 2.3. Ethical Status of Organoids and Organ-on-a-Chip Models

With continuous technological advancement, the biomimetic sophistication and functional complexity of organoids and organ-on-a-chip models have progressively increased, sparking debates about their ethical status—particularly prominent in brain organoid research. Brain organoids were initially developed primarily to simulate the structural processes and molecular mechanisms of brain development. However, recent studies have demonstrated that during their in vitro development, these organoids can exhibit electrophysiological activity resembling brain rhythms. Under specific conditions, they can even respond to light stimulation and display synchronized neural network discharge patterns [37]. While these findings do not prove that brain organoids possess full human consciousness or subjective experience, the “brain-like activity” they exhibit compels the ethics community to re-examine the moral status of brain models [38].

Currently, the prevailing international ethical consensus holds that brain organoids do not yet possess consciousness. However, given that brain function involves core human characteristics such as emotion and volition, researchers must remain highly sensitive to the potential risks arising from technological advancements. Particularly, if organoids exhibit brain-like functions such as spontaneous electrical activity, regionalized differentiation, and synapse formation, and subsequently develop the capacity to respond to external stimuli or show tendencies toward self-organization of neural signals, they may reach an ethically significant threshold. This potential “quasi-consciousness” risk necessitates that researchers and ethics committees adopt a precautionary principle when designing and reviewing experiments, clearly defining the boundaries of technology application and establishing evaluation criteria [39]. For example, Quadrato et al. demonstrated photosensitive neural networks and coordinated activity in human cerebral organoids, findings that subsequently stimulated ethical debate regarding consciousness-related properties and the moral status of cerebral organoids [37].

Although no international consensus currently exists regarding a definitive ethical threshold for brain organoids, a preliminary framework may be developed from three complementary perspectives. First, structural considerations focus on the degree of neural organization and complexity achieved by the organoid. Second, functional considerations focus on whether the organoid exhibits increasingly sophisticated patterns of neural activity that may be relevant to consciousness or morally significant forms of information processing. Third, application-based considerations recognize that ethical concerns may vary according to the intended use of the organoid, such as disease modeling, transplantation, or integration into animal hosts. Rather than relying on any single criterion, future governance frameworks should incorporate structural, functional, and application-based assessments to identify ethically significant developments and ensure proportionate ethical oversight [38,39].

In contrast, non-neural organoids typically pose lower ethical risks. However, as their functional complexity increases, new ethical questions emerge. For instance, liver organoids induced in vitro can express typical hepatocyte markers and exhibit key drug metabolism functional activity [40]. Heart organoids can maintain autonomous beating rhythms and respond to drug stimuli, enabling early screening for cardiac toxicity [41]. Although these models do not involve consciousness, their “life-like behaviors” may influence public perception and regulatory policies. If such models are applied to transplantation research, personalized medicine, or commercial services, they raise ethical concerns regarding donor rights, data privacy, and transparency in commercial exploitation. Balancing scientific innovation with preventing the misuse of biological models represents a core challenge for non-neural organoid research.

Consequently, the rapid advancement of brain organoids and other functional organoids, while significantly propelling progress in life sciences and drug innovation, also necessitates a shift in ethical safeguards from reactive responses to proactive protection. This transition relies both on advances in electrophysiological monitoring technologies and timely adjustments to ethical frameworks—including their design, expert composition, and dynamic evaluation mechanisms. Maintaining a balance between scientific progress and ethical responsibility is essential for the sustainable development of organoid and organ-on-a-chip research.

### 2.4. Ethical Risks of Chimeras

Human–animal chimera research enables the introduction of human cells or their derivatives into animal hosts, thereby reconstructing or simulating the developmental processes of human tissues and organs. This approach represents a crucial technical pathway for advancing MPS toward higher-level in vivo biomimetic development [42]. However, this research direction has also raised extensive ethical concerns, particularly in the context of integrating human cells with animal systems, potentially exerting profound impacts on species boundaries, ideology, and reproductive safety [43].

For instance, implanting human-derived neurons or brain organoids into animal brain regions may alter animal behavior, cognition, or consciousness states [44]. Such interventions are particularly likely to affect brain function during embryonic or neonatal stages when brain development is incomplete. Furthermore, injecting human germ cells into animals may breach species boundaries, raising ethical concerns about “interspecies reproduction.” This further-challenges traditional distinctions of human identity and biological boundaries, introducing ethical risks associated with the expression of human characteristics in non-human animals [43]. A representative example is the transplantation of human brain organoids into animal brains, which has prompted discussion regarding the potential alteration of cognition, moral status, and animal welfare in recipient animals [44].

These ethical risks may arise in several specific forms. First, the integration of human neural cells or brain organoids into animal brains may raise concerns regarding altered cognition, consciousness-related capacities, and the moral status of the resulting animals [44]. Second, the contribution of human cells to animal germlines may challenge reproductive boundaries and species integrity [43]. These concerns have prompted increasing discussion regarding appropriate ethical oversight, animal welfare protections, and regulatory governance of human–animal chimera research. Legal and regulatory approaches differ across jurisdictions. In the United States, certain categories of chimera research involving neural or germline contribution are subject to heightened ethical scrutiny and funding restrictions [45]. In Europe, oversight is generally addressed through specialized ethical review and national regulatory mechanisms, whereas China’s recent guidelines identify brain organoid and chimera research as areas requiring strengthened ethical review and risk assessment [7].

Multiple countries worldwide have progressively established ethical review mechanisms for chimera research, requiring comprehensive risk assessments prior to study initiation. These assessments must encompass anticipatory evaluations and response strategies concerning animal welfare, species boundaries, and potential expression of human characteristics. Numerous international policy documents and ethical research reports emphasize the need for extreme caution in chimera studies. In a 2021 report, the U.S. National Academies of Sciences, Engineering, and Medicine highlighted that neural chimera research may raise numerous concerns. It recommended closely monitoring developments in this field, encouraged pilot studies, and emphasized the need for rigorous observation of animal behavior. China’s National Committee on Bioethics for Science and Technology, through its Subcommittee on Life Sciences Ethics, issued the Ethical Guidelines for Human-Derived Organoid Research. These guidelines stipulate that when conducting organoid-chimera studies, researchers must carefully select the quantity of human cells for transplantation, rigorously assess ethical risks, adhere to relevant regulations such as the Ethical Guidelines for Human-Nonhuman Animal Chimera Research, and respect the welfare of experimental animals [7].

Overall, organoid-chimera technology indeed presents new opportunities for constructing humanized xenografts and offers a potential pathway to alleviate the global organ shortage. However, the accompanying ethical challenges cannot be overlooked. In particular, research involving human–animal chimeras gives rise to concrete concerns regarding species boundaries, moral status, and animal welfare. When human neural cells are introduced into non-human animal brains, uncertainties emerge as to whether and to what extent human-like cognitive capacities might develop, thereby challenging conventional distinctions between humans and animals and complicating moral evaluation and ethical responsibility [9]. The lack of clearly defined thresholds for ethically significant neural integration makes it difficult to determine when such experiments warrant heightened oversight or restriction. Moreover, the potential contribution of human cells to the reproductive systems of host animals raises concerns about unintended germline transmission and the creation of biologically ambiguous lineages, outcomes that are widely regarded as ethically impermissible across regulatory frameworks. These biological uncertainties also have direct implications for animal welfare, as chimeric animals with altered neural or physiological characteristics may experience atypical forms of distress or suffering that are not adequately addressed by standard welfare assessment protocols [45]. Together, these challenges underscore the need for more nuanced ethical review mechanisms that go beyond species-based categorizations and explicitly account for the moral significance of neural and reproductive integration in chimera research. Currently, international regulatory trends increasingly emphasize transparency, auditability, and traceability in research processes, aiming to achieve a dynamic equilibrium between scientific innovation and ethical boundaries. This approach not only promotes the healthy development of scientific research but also better safeguards the fundamental rights of both humans and animals, fostering sustainable progress in science and technology.

To move beyond general risk awareness, several international ethics bodies have proposed the establishment of tiered ethical boundaries for chimera research based on the likelihood of human cell contribution to neural and reproductive systems [9]. Under this approach, studies involving limited integration of human cells into non-neural tissues for disease modeling or drug testing are generally considered ethically acceptable following standard ethical review procedures. In contrast, experiments involving substantial human neural cell contribution to the central nervous system, particularly during embryonic development or in non-human primates, may require enhanced ethical oversight due to concerns regarding potential alterations in cognition, consciousness, or behavior [44]. Even stricter restrictions are commonly recommended for studies involving the contribution of human cells to animal germlines or reproductive tissues, given the possibility of generating human gametes or transmitting human genetic material across generations [45]. Therefore, future governance frameworks for MPS-related chimera research should adopt a risk-tiered regulatory strategy that differentiates between acceptable, restricted, and prohibited applications according to the degree of neural and reproductive integration, thereby providing clearer ethical boundaries while preserving scientific innovation.

## 3. Strategies for Addressing Ethical Challenges of Microphysiological System Technology

### 3.1. Promote the Construction of a Dynamic Ethical Governance Mechanism

As the application of MPS technology continues to expand from short-term in vitro experimentation toward long-term disease modeling, personalized medicine, and complex human–animal chimera research, traditional one-time ethical review mechanisms are increasingly recognized as insufficient. This challenge has been explicitly acknowledged across major regulatory jurisdictions. In the United States, ethical oversight of advanced cellular models is gradually shifting toward a lifecycle-based governance approach, in which Institutional Review Boards (IRBs) and funding agencies emphasize continuous monitoring, protocol amendments, and re-evaluation of risk as research objectives, model complexity, or downstream applications evolve. NIH policy discussions on dynamic consent and secondary use of biospecimens highlight the need to reassess donor expectations and ethical risks over time, particularly for reusable and expandable systems such as iPSC-derived MPS platforms [36].

In Europe, although ethical review remains institutionally anchored, the concept of dynamic governance is reinforced through data protection law. Under the GDPR, accountability, traceability, and purpose limitation requirements effectively mandate ongoing ethical responsibility throughout the entire data lifecycle, rather than at the point of initial approval [29]. This regulatory logic aligns with emerging ethical discussions around sustained informed consent and adaptive oversight for long-term organoid and MPS research. China has similarly begun to emphasize continuous supervision through post-approval oversight, project registration, and periodic inspection mechanisms under its ethics governance system for human-derived advanced biomedical research, reflecting a convergence toward dynamic ethical governance despite differences in administrative structure. Collectively, these developments indicate a shared international recognition that ethically sustainable MPS research requires governance mechanisms capable of adapting to scientific uncertainty, model evolution, and prolonged data utilization [9].

### 3.2. Strengthen Mutual Recognition of International Standards and Regulatory Coordination

Beyond institutional ethics review, international regulatory fragmentation remains a major barrier to the global development and validation of MPS technologies. Significant differences persist among countries with respect to ethical requirements for cell sourcing, validation criteria for organ-on-chip systems, standards for data interoperability, and regulatory treatment of human–animal chimeras [46]. As noted by Ito et al., the absence of harmonized international standards for defining the “context of use” of MPS—from model design to validation—limits cross-study comparability and impedes regulatory acceptance of generated data [47].

In response, international organizations have increasingly assumed a coordinating role. The OECD has promoted standardized validation frameworks through its Guidelines for the Testing of Chemicals and the Good In Vitro Method Practices (GIVIMP), aiming to facilitate cross-border data acceptance of New Approach Methodologies (NAMs) [48]. Similarly, ISO/TC 276 has focused on establishing technical standards for cell characterization and MPS interoperability to reduce fragmentation in industrial applications [49]. In the United States and Europe, regulatory agencies such as the FDA and EMA are actively incorporating NAMs into their strategic frameworks. For instance, the EMA’s 3Rs Working Party has intensified efforts to qualify alternative methods for regulatory use, emphasizing a shift from animal models to human-relevant tools [50]. Meanwhile, China acts through the NMPA, aligning domestic technical guidelines with international trends while emphasizing biosecurity and centralized data oversight. These parallel efforts suggest that future ethical governance will depend on a “multi-layered” international coordination mechanism that synchronizes ethical review, technical validation, and regulatory recognition [51].

### 3.3. Promoting Interest Sharing and Public Participation

The mechanism of benefit sharing and public participation should be placed at the core of governance. MPS research heavily relies on human cells, patient tumor tissues, and multiple data resources, and its results may be transformed into high-value drugs or precision medical products. To enhance the social trust foundation of scientific research, research institutions and enterprises should explore a fair benefit distribution mechanism between sample/data donors and commercial benefits. For example, in the initial informed consent stage, it is necessary to clearly state the future commercial use, whether the donor has the right to receive product benefits, and whether they participate in the decision-making process. In addition, building a public platform for ordinary people and patients to participate in public discussions on the expected impact of MPS technology will help strengthen research transparency and social responsibility [52]. This shift from “expert-led” to “expert + public co-governance” is the key to the sustainable development of MPS ethics. Finally, open data and standardized infrastructure are also important directions for future development. Although the MPS platform is increasingly generating large-scale genome, transcriptome, and organ-to-organ interconnected data, its potential value has not been fully realized due to the lack of standards, inconsistent data formats, incomplete privacy protection, and cross-border circulation mechanisms. If MPS research can make breakthroughs in material standardization, model validation standards, data specifications, etc., it will lay a solid foundation for regulatory acceptance and industrial application [53]. The future governance directions and key implementation mechanisms are summarized in Table 3.

## 4. Conclusions

The rapid advancement of MPS technology is profoundly reshaping paradigms in disease modeling, drug development, and precision medicine. However, its high biological complexity and human relevance have also brought ethical, legal, and social governance issues to the forefront. This review systematically examines the core ethical challenges facing MPS—ranging from controversies over the sourcing of human stem cells and privacy risks associated with individual genetic data, to the ambiguous moral status of organoids (particularly brain organoids), and the challenges human–animal chimera research poses to species boundaries. Concurrently, fragmented regulatory frameworks globally, along with the lack of technical standards and validation systems, further hinder the translation of MPS from the laboratory to industrialization and clinical application. To address these challenges, we argue that the future of MPS depends not only on technological breakthroughs but also on the establishment of a corresponding multi-level, dynamic, and internationally coordinated governance system. First, ethical review should evolve from a one-time approval model toward lifecycle-based dynamic oversight, establishing continuous evaluation mechanisms that accommodate long-term studies, sample reuse, and model evolution. Second, there is an urgent need to strengthen international standard harmonization and regulatory mutual recognition. Organizations such as ISO and OECD should promote the standardization of MPS model validation, data formats, and metadata to reduce technical barriers. Third, benefit-sharing and public engagement must be integrated into the core of governance. Informed consent procedures should clearly address potential commercial use, and equitable return mechanisms should be explored, while public consultations and transparent communication should enhance social trust and research legitimacy. Finally, efforts should be made to build open and well-structured data and model infrastructures that facilitate data sharing and scientific collaboration under privacy protection, thereby laying the foundation for regulatory acceptance.

Importantly, not all ethical challenges discussed in this review are at the same stage of development. Certain issues, including informed consent, donor privacy, data governance, commercialization of biological materials, and cross-border sharing of human-derived data, are already encountered in current MPS research and therefore require immediate governance attention. In contrast, concerns related to the potential emergence of consciousness in advanced brain organoids, substantial cognitive humanization in human–animal chimeras, and other ethically transformative biological capabilities remain largely speculative and are more appropriately addressed through anticipatory governance and ongoing ethical assessment. Distinguishing between current and future-oriented challenges may help prioritize regulatory actions while maintaining preparedness for emerging developments in MPS research.

For researchers, advancing MPS requires not only asking “what can we do?” but also continually reflecting on “how can we do it responsibly?” and “who will benefit and who will bear the risks?” In this sense, the sustainable development of MPS is not merely a scientific or technological issue but also a systemic project involving the reconstruction of ethical governance and the renewal of the social contract. Only through multidisciplinary dialogue and collaborative engagement among scientists, ethicists, policymakers, and the public can we promote scientific frontiers while upholding ethical principles, ultimately realizing the vision of MPS technology serving human health and social well-being.

Looking ahead, emerging technological convergence may introduce a new generation of ethical and governance challenges for MPS. In particular, the integration of MPS with artificial intelligence (AI), machine learning, and gene-editing technologies has the potential to significantly expand the capabilities of these platforms while simultaneously increasing ethical complexity [59]. AI-assisted analysis of large-scale MPS datasets may improve disease prediction, drug screening, and personalized medicine; however, it also raises concerns regarding algorithmic transparency, data ownership, bias, accountability, and the explainability of decision-making processes [59]. Likewise, the incorporation of gene-editing technologies such as CRISPR-Cas systems into MPS models enables precise disease modeling and functional validation but may introduce additional ethical questions concerning genetic manipulation, dual-use research risks, and the creation of increasingly complex engineered biological systems. As these technologies become more deeply integrated, future governance frameworks should move beyond technology-specific regulation and adopt adaptive, interdisciplinary approaches capable of addressing the ethical implications arising from the convergence of AI, gene editing, and advanced human-relevant biological models [59]. Anticipatory governance and continuous ethical assessment will therefore be essential to ensure that innovation proceeds in a socially responsible and trustworthy manner.

## Figures and Tables

**Table 1:** Overview of MPS-related models, applications, advantages, limitations, ethical concerns, and governance considerations.

Model	Ethical Relevance in MPS Research	Key Ethical and Regulatory Concerns	Governance Considerations
Organ-on-a-chip	Involves human primary cells, stem-cell-derived cells, organoids, and multi-cellular microfluidic platforms, thereby raising issues related to human cell sourcing, consent, data use, and regulatory validation	Human cell sourcing, complex physiological systems, data privacy, boundaries of application scope, standardization and validation, regulatory acceptance of MPS-derived evidence	Establish clear consent procedures for human-derived cell use, implement privacy-compliant data governance; define context of use, develop model validation and quality standards
Organoid	Uses self-organizing human-derived stem cells or patient-derived tissues to model development, disease, and personalized responses	Human cell sourcing, repeated and multi-lineage differentiation, scope of donor consent, reuse and long-term culture, personal and genetic data protection, commercialization and benefit sharing, ethical concerns in brain organoids	Implement dynamic consent and life-cycle ethical review, establish risk-tiered oversight for brain organoids and chimera-related studies; ensure animal welfare, transparency in commercialization, and responsible data sharing

**Table 2:** Comparison of International Ethical Regulatory Frameworks.

Region	Main Regulatory Bodies	Primary Frameworks and Documents	Regulatory Focus and Scope	Ethical Governance Features
United States	National Institutes of Health (NIH); Food and Drug Administration (FDA)	Health Insurance Portability and Accountability Act (HIPAA, 1996) [28]; NIH Guidelines for Human Stem Cell Research (2009) [36];	The NIH guidelines specify the scope of federally funded human embryonic stem cell (hESC) research and require explicit donor informed consent [9]. HIPAA regulates medical and research data privacy, de-identification, and lawful secondary use [28].	Research governance centers on federal funding control and tiered privacy protection; Institutional Review Boards (IRBs) oversee ethical compliance.
Europe	European Medicines Agency (EMA); European Group on Ethics in Science and New Technologies (EGE); European Commission; Human Fertilisation and Embryology Authority (HFEA, UK)	General Data Protection Regulation (GDPR, 2018) [29]; EGE Opinions on Ethics of New Technologies (2021) [32]; Human Fertilisation and Embryology Act (UK, 1990; amended 2008) [33]	GDPR ensures strict data protection and cross-border data management [29]; EGE provides ethical guidance on emerging life science technologies to the European Commission [32]; in the United Kingdom, the Human Fertilisation and Embryology Acts established the “14-day rule” and created the HFEA as an independent regulatory authority [33].	Ethics governance integrates EU-level data protection with diverse national regulatory approaches across Europe, emphasizing transparency, public engagement, and precautionary oversight.
China	Ministry of Science and Technology (MOST); National Medical Products Administration (NMPA); National Science and Technology Ethics Committee	Ethical Guidelines for Research on Human Embryonic Stem Cells (2003) [30]; Regulations on the Administration of Human Genetic Resources (2019) [31]; Ethical Guidelines for Human Organoid Research (2025) [7]	Explicitly prohibits reproductive use and interspecies reproductive attempts [30]; mandates ethical review and registration [7]; strengthens informed consent and oversight for organoid and chimera studies [7].	Establishes a dual-layer governance model combining institutional self-review and governmental registration; emphasizes continuous oversight and social responsibility throughout the research process.

**Table 3:** Future Governance Directions and Key Mechanisms for MPS.

Governance Direction	Key Stakeholders	Objective	Implementation Pathways/Actions
Advancing Dynamic Ethical Review and Continuous Oversight	Institutional Ethics Committees, Funding Agencies, Regulatory Authorities	Ensure ongoing oversight of long-term sample use, model evolution, and chimera-related risks	Establish periodic or rolling ethical review mechanisms; promote dynamic consent platforms [54]
Promoting International Standard Harmonization and Regulatory Coordination	ISO, OECD, WHO, ICH, National Regulators	Harmonize validation standards and facilitate cross-border data interoperability and model recognition	Utilize ISO/CEN, OECD, and WHO platforms to develop MPS context-of-use validation processes and metadata standards [55]; integrate NAMs into ICH and other international guidelines
Establishing Benefit-Sharing and Fair Compensation Mechanisms	Research Institutions, Industry, Ethics Committees, Patient Organizations	Protect sample/data donor rights and enhance public trust	Define commercial use and benefit-sharing terms in initial consent forms; explore community- or patient-based benefit-sharing models [52]
Enhancing Public Engagement and Transparent Governance	Research Institutions, Funding Agencies, Patient/Public Organizations, Media	Increase societal acceptance and foster participatory governance	Build public consultation platforms [56]; include patient representatives in ethics committees; regularly disclose research progress and risk assessment reports
Accelerating Open Data and Metadata Standardization	Research Consortia, Data Standard Bodies, Journals, Funding Agencies	Improve data reproducibility, sharing, and regulatory acceptance	Develop standardized MPS metadata schemes; unify data formats; establish privacy-compliant controlled-access open databases [57]
Promoting Model Validation and Regulatory Acceptance	Industry, Regulatory Authorities (FDA/EMA/NMPA), Third-Party Validation Centers	Enable validated MPS to be adopted in pharmaceutical and chemical regulatory frameworks	Develop standardized validation toolkits; conduct multi-center ring-trials; promote inclusion of NAMs guidance under the ICH framework [58]

## Data Availability

No new data were created or analyzed in this study. Data sharing is not applicable to this article.
